# Effects of lower limb joint manipulation on squat strength and ankle range of motion: a sham-controlled double-blind randomised cross-over trial

**DOI:** 10.1186/s12998-026-00659-7

**Published:** 2026-06-19

**Authors:** Reneigh Morley-Hart, Barrett Losco, Alasdair R. Dempsey, G. Gregory Haff, Sasha L. Aspinall

**Affiliations:** 1 School of Allied Health, Murdoch University, Perth, Australia; 2 Precision Medicine Center, Health Futures Institute, Murdoch University, Murdoch, WA Australia; 3 Strength and Power Research Group, School of Medical and Health Sciences, Edith Cowan University, Perth, Australia; 4 School of Health & Society, University of Salford, Greater Manchester, UK

**Keywords:** Joint manipulation, Back squat, Dorsiflexion

## Abstract

**Background:**

The back squat is a commonly prescribed exercise for developing lower body strength. The ability to squat can be affected by reduced ankle dorsiflexion range of motion (DF-ROM), leading to limited squat depth and dysfunctional compensatory movement patterns.

**Objective:**

To investigate the effect of high-velocity low-amplitude lower limb manipulations on 1 repetition maximum (1RM) back squat strength, sagittal plane ankle kinematics during a squat, and active DF-ROM in individuals with asymptomatic deficits in DF-ROM.

**Methods:**

Twenty-six enrolled; Twenty-four analysed resistance-trained participants with asymptomatic deficits in DF-ROM participated in this double-blind randomised sham-controlled cross-over trial. Interventions involved high-velocity low-amplitude joint manipulations targeting ankle DF-ROM, and a sham which mimicked the manipulations using a mechanical drop mechanism. The primary outcome was a change in back squat 1RM between baseline to post-intervention, with secondary outcomes being maximal ankle DF-ROM during the 1RM squat using 3D motion capture, and change in DF-ROM during the weightbearing lunge test.

**Results:**

There was no statistically significant difference between interventions for back squat 1RM (*p* = 0.27). From baseline, the 1RM increased by 4.75 kg (3.6%) following manipulation and 2.75 kg (2.1%) following the sham. There were no statistically significant differences between interventions on maximal DF-ROM during the back squat (*p* = 0.24) and no statistically significant differences in ankle DF-ROM immediately following either intervention (*p* = 0.39).

**Conclusion:**

Based on these results a single session of high-velocity low-amplitude manipulations targeting ankle DF-ROM does not significantly improve 1RM back squat strength compared to sham, and does not influence short-term ankle DF-ROM.

*Trial registration* The study was pre-registered with ANZCTR (ACTRN12622000811707).

## Background

The back squat is a fundamental resistance training exercise used for improving sports performance and activities of daily living [1–4]. This exercise is commonly integrated into a resistance training program when attempting to increase lower limb muscular strength, improve muscular coordination in the lower limbs, and engage muscles across a wide range of joint movements [1, 3]. Key benefits of increasing squat strength are its impacts on the elastic properties of muscles and tendons involved in the stretch–shortening cycle, and improved rate of force development, allowing for quicker and more efficient utilisation of the stretch–shortening cycle [1, 3, 5]. Increases in maximal back squat strength have also been associated with improvements in sports performance measures, such as sprint speed, vertical jump displacement, and weightlifting [6–9]. Additionally, increases in squat strength may be related to reduced risk of lower limb injury [10].

Full-depth squats, characterised by descending to a position where the hip joint is below the knee joint, are preferred for increasing squatting strength and promoting muscle growth [11–13]. It is a symmetrical movement and it is important to consider range of motion (ROM) during the exercise, as ROM is a significant predictor of strength [1, 14] and squat performance [15]. Achieving a full-depth squat requires adequate ROM in the ankle, knee and hip. Among these, the ankle complex is crucial for performing the squat movement [16] due to its role in dorsiflexion and plantarflexion, which occurs primarily at the talocrural joint and a small amount at the subtalar joint [17].

Normal active ankle dorsiflexion range of motion (DF-ROM), when measured via the weightbearing lunge test (WBLT), is typically > 44° [18]. In contrast, the dynamic kinematic requirement for ankle DF-ROM during a parallel back squat typically reaches 23.4—30.8° [15, 19]. Limited DF-ROM can impact squat depth, lead to muscle inhibition, and increase the risk of injuries [15, 20, 21]. Addressing ankle mobility may be important for improving back squat biomechanics and performance in some individuals, and joint manipulation is one intervention that can potentially improve ankle mobility [22]. In an asymptomatic, resistance trained population, any potential acute improvement in mobility is theorised to stem from mechanical and arthrokinematics changes such as, increased joint play, improved posterior talar glide, and altered capsular compliance, rather than the relief of neurophysiological muscle inhibition. These arthrokinematics optimisations may subsequently facilitate a more efficient length-tension relationship and enhance motor unit recruitment during maximal exertion [23].

Studies on the effect of lower limb manipulation on DF-ROM show varying results [24–31]. Single talocrural joint HVLA manipulation may not significantly impact DF-ROM in asymptomatic individuals [26, 28, 31]. Similarly, a single session of proximal or distal tibiofibular joint manipulations may not improve DF-ROM in those with a history of chronic ankle instability or sprained ankles [27, 29]. However, multiple talocrural joint manipulations during a single session in those with chronic ankle instability may significantly increase DF-ROM [25, 32]. Additionally, manipulations to either multiple joints [24] or over multiple treatment sessions [26, 29, 30] may be more likely to increase DF-ROM in asymptomatic and symptomatic participants. One of the above studies compared to sham interventions [29].

A small number of studies examining lower limb manipulation have reported positive effects on muscle activation [33, 34] and functional performance measures including the speed-test and hop test [35]. Additionally, full body chiropractic manipulation, including extremity manipulation, have resulted in improved stepping reaction time functional tests [36]. Conversely, Hedlund et al. [37] have reported no statistical difference in vertical jump height following lower limb manipulations compared to a sham. To the authors knowledge there are prior studies that have directly investigated the effect of lower limb manipulations on squat performance or mechanics.

Therefore, the primary purpose of this study was to examine the effects of a single session of HVLA manipulations to multiple lower limb joints on high-bar back squat strength in individuals with limited ankle DF-ROM. The secondary purpose was to examine change in DF-ROM during the back squat and maximal active DF-ROM.

## Methods

This study was a double-blind randomised sham-controlled cross-over trial. Ethics approval was acquired from Murdoch University Human Research Ethics Committee (Approval 2022/085) and the study was pre-registered with ANZCTR (ACTRN12622000811707).

### Participants

Participants consisted of a convenience sample of physically active adults who had limited DF-ROM in at least one ankle. Inclusion criteria were a) 18–55 years of age, b) at least one ankle with a DF-ROM measure < 44.0°on the WBLT, c) able to squat to a depth in which the thighs are parallel to the floor (inguinal fold below the apex of the knee), d) able to squat at least one times body weight for a one repetition maximum (1RM), e) 12-month history of resistance training including back squats averaging at least one session per week, and f) squatting at least once per week for eight weeks prior to screening.

A static WBLT measure of < 44.0° was chosen as it serves as a validated clinical threshold for underlying ankle stiffness that may drive compensatory kinematic strategies during dynamic squatting tasks [18]. While the allowance of heeled shoes in this study reduces the absolute dorsiflexion requirement to reach parallel depth [38, 39], this baseline stiffness is theorised to still influence the kinetic chain under maximal load. The criterion of being able of squat one times body weight was deliberately selected to minimise the confounding influence of novice motor learning and to improve safety during maximal testing. However, we acknowledge an inherent tension this creates: selecting a well-trained cohort increases the likelihood of a physiological ceiling effect, which theoretically reduces the chance of detecting acute, rapid strength gains following a single manual therapy intervention [40].

Exclusion criteria were (a) any contraindication to extremity manipulation, (b) history of neurological diseases, rheumatoid diseases, or osteoarthritis of lower limbs or spine, (c) injury within the past six months to the ankle, knee or hip including sprain, strain, or fracture, (d) major surgery to the lower limbs in the past 12 months, (e) surgery that included internal fixations in the lower limbs, (f) received any joint manipulation to the lower limbs in the past six weeks, (g) impaired foot and ankle function indicated by a score of less than 80% on the Foot and Ankle Disability Index (FADI) questionnaire, and (h) any contraindications to exercise indicated by a fail on stage 1 of the Exercise & Sports Science Australia Adult Pre-Exercise Screening System. In addition, female participants were excluded during the follicular phase of their self-reported menstrual cycle (during menstrual bleeding) since there is an increased risk of injury due to ligament laxity at this time [41], or if they were pregnant.

### Sample size

A power calculation was performed using G*Power v3.1 based on Taskin et al. [42]. An effect size of 0.62 was calculated based on the 1RM back squat weight at baseline and after a lower limb tape intervention. With alpha at 0.05 and 80% power, an a priori sample size of 23 was determined. To allow for 10% dropout, the final target sample size was 26. While a lower limb tape intervention is different to the intervention in our study, we were unable to identify a study with the same primary outcome and more closely aligned interventions for the power calculation. Hence, this calculation was an approximation based on the best available comparison study we were able to identify.

### Procedure

The study was completed at the Murdoch University Performance Laboratory and involved three sessions. The first session involved informed consent, screening for inclusion, familiarisation, and baseline testing. Three to five days later, the first treatment session occurred, followed by the second treatment session seven to eight days later. The participants were assigned to a randomised intervention sequence at the first treatment session. Participants were required to abstain from any exercise 24 h prior to each session.

### Familiarisation session

Participants completed informed consent and inclusion questionnaires, followed by recording height on a wall-mounted stadiometer (Seca 206, SECA, Hamburg, Germany) and weight measurements on a flat scale (Seca 762, SECA, Hamburg, Germany). Next, the weightbearing lunge test (WBLT) used a digital goniometer an EasyAngle® (Meloq AB, Sweden), with an accuracy of ± 1° to measure ankle DF-ROM in accordance with the method outlined by Dill et al. [18]. This method has good intra-rater (ICC = 0.98, 95% CI = 0.93–0.99) and inter-rater reliability (0.97, 95% CI = 0.90–0.99) [43]. The WBLT was performed at each session where the examiner took 3 measurements per leg and the mean of the measures for each leg were then used for data analysis. Participants completed a standardised full body raise, active, mobilise and potentiate (RAMP) warm-up protocol [44]. This consisted of 10–15 min of body weight walking lunges, side squats, ankle grabs, and jumping movements. Ankle DF-ROM was then re-measured after the warm-up using the WBLT.

**Fig. 1:**
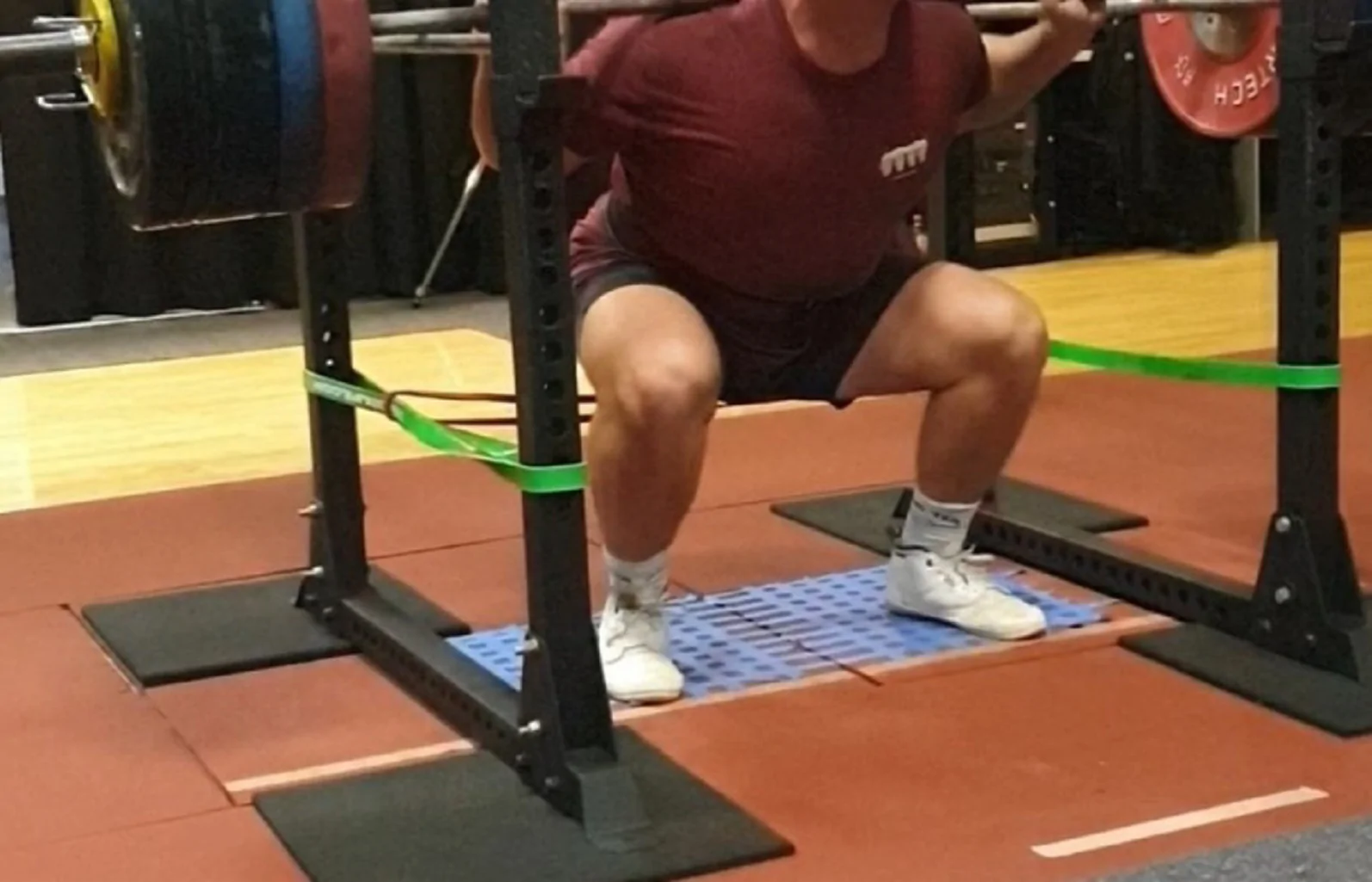
Squat rack, foot position tape and squat height

Finally, baseline 1RM during a free weight high-bar back squat was measured using a 20 kg Armourtech barbell inside of a body-solid power rack and following the procedure outlined by Banyard et al. [45]. Participants were allowed to wear their normal weightlifting shoes (limited to a one-inch heel lift), weight belt, and knee sleeves, which were required to be consistent at all sessions. Participants may be accustomed to their usual gear, and this study aimed to create an ecologically valid squat environment and detect changes to strength under each participant’s normal squatting conditions [46, 47]. We acknowledge that this standardisation also introduced potential effect modifiers. Specifically, the use of heeled shoes alters lower limb kinematics and reduces the absolute ankle DF-ROM required to reach parallel depth, an interaction that is explored further in the discussion [46, 47]. The squat depth minimum was recorded and photographed Kotani et al. [48] (Fig. 1) for replication at subsequent sessions. Foot position was recorded and standardised using a tape grid and a picture taken to replicate at subsequent sessions.

### Treatment sessions

**Fig. 2:**
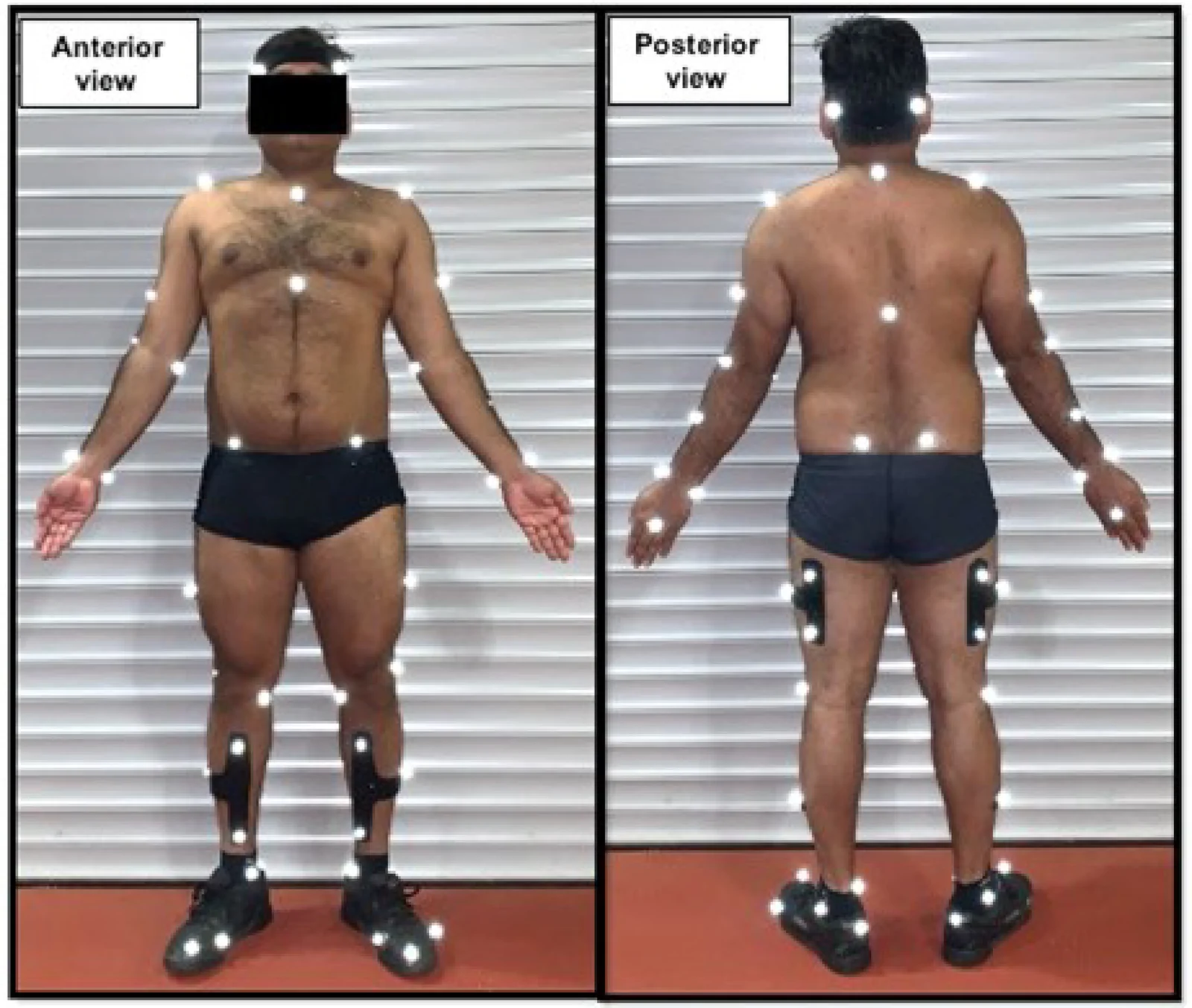
Placement of retro-reflective markers on the participants body for 3D motion analysis

At each treatment session, participants completed a WBLT and the RAMP warm-up, followed by a randomised intervention. One minute post-intervention, the WBLT was performed again, followed by a 1RM high-bar back squat based on the methods of Banyard et al. [49], which is based on the 1RM established at the familiarisation session and has the advantage of being less fatiguing while still accurate. Kinematic data were collected during all repetitions at 70%, 80%, 90% and 100% 1RM squats using a 12-camera motion capture system (Oqus3 + cameras [Qualisys AB, Gothenburg, Sweden], Qualisys Track Manager software) [50]. Retro-reflective markers were placed on specific anatomical landmarks (Fig. 2) [51]. At the completion of treatment session two, participants completed a debrief and a blinding questionnaire.

### Randomisation

Participants were randomised to intervention sequence 1 or 2 by a computer-generated random sequence (https://www.random.org/) of 1’s and 2’s, each contained in sequentially numbered, sealed, opaque envelopes which were opened by the therapist delivering interventions just prior to the intervention at treatment session one. Sequence 1 involved lower limb manipulations at treatment session one and sham manipulations at treatment session two, while sequence 2 was the opposite.

### Blinding

The participant and outcome assessor were blinded to the interventions, and therapists were unaware of the participant’s outcomes. Participants were unaware they might receive a sham intervention and were told the study was comparing two manipulation interventions. On completion of the final session participants were debriefed about the nature of the study and asked to complete a blinding questionnaire. The blinding questionnaire asked whether, at the time of each treatment, they believed they received a real treatment, with response options of “yes”, “no”, or “I wasn’t sure.”

### Interventions

Participants received either bilateral lower limb HVLA manipulations or bilateral sham manipulations at each treatment session, delivered by one of two chiropractors (SA or BL), each with at least eight years of clinical experience. The interventions are described in additional detail in Supplementary File 1.

**Fig. 3:**
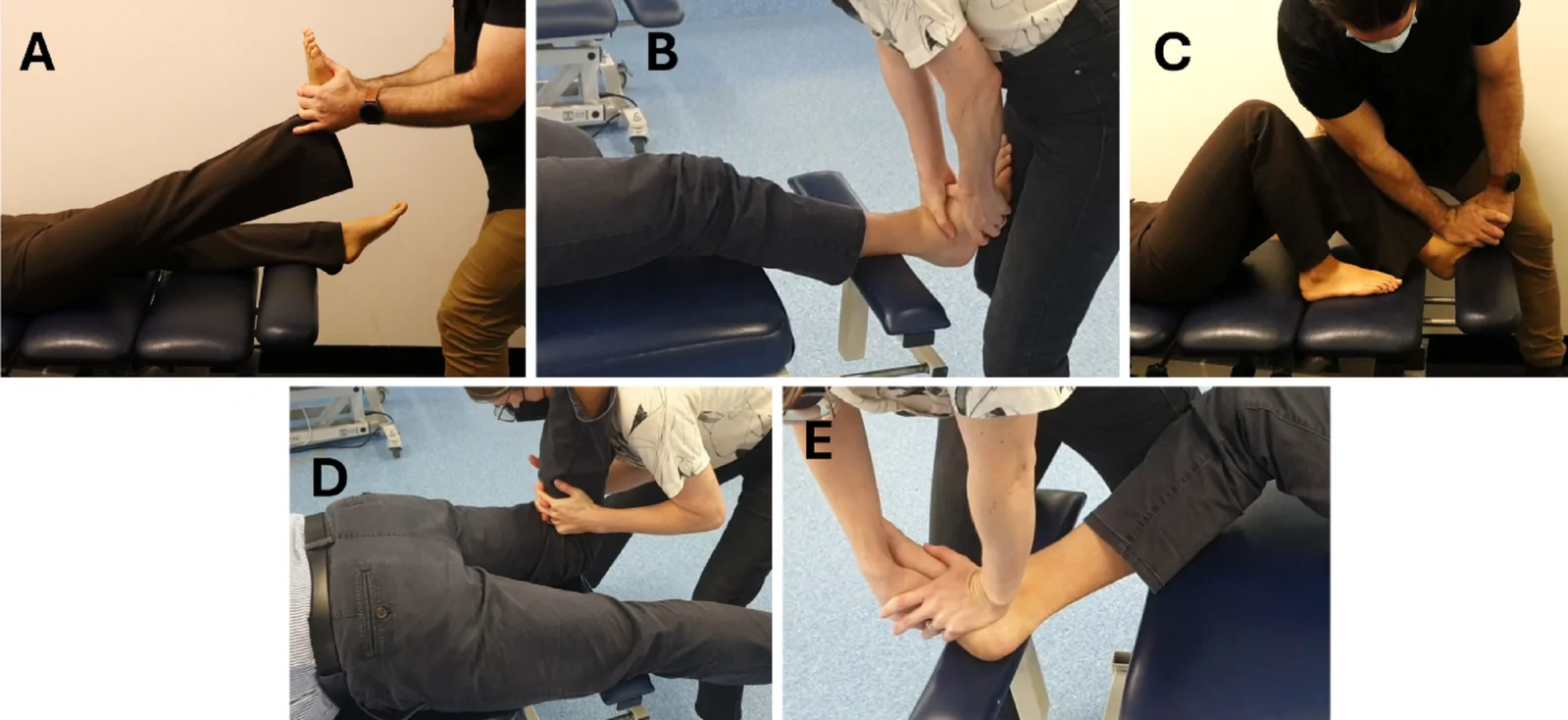
High-velocity low-amplitude manipulations. **a** Talocrural joint long-axis distraction, **b** Talocrural joint anterior to posterior, **c** Tarsometatarsal joint anterior to posterior, **d** Proximal Tibiofibular joint posterior to anterior, **e** Subtalar joint medial to lateral

The HVLA manipulations were performed bilaterally targeting the talocrural, subtalar, tarsometatarsal, and proximal tibiofibular joints, shown in Fig. 3. A maximum of three attempts at each joint was allowed, based on perceived success of each attempt, determined subjectively by the therapist, and not necessarily based on an audible cavitation. The manipulations were collectively intended to encourage mobility and especially dorsiflexion of the ankle/foot complex. The therapists were trained in a single 30-min session prior to the study commencing to improve consistency for both interventions. For the sham, therapists were specifically trained to use a broad, non-joint-specific contact and to deliver the mock thrust directly into the table with subjectively similar speed and force. The therapists also completed a form immediately after each intervention to indicate how many attempts at each manipulation or sham were made, which also served to ensure interventions were delivered as intended. These forms were held by the therapists until after all interventions were delivered to avoid unblinding the assessor (RM).

**Fig. 4:**
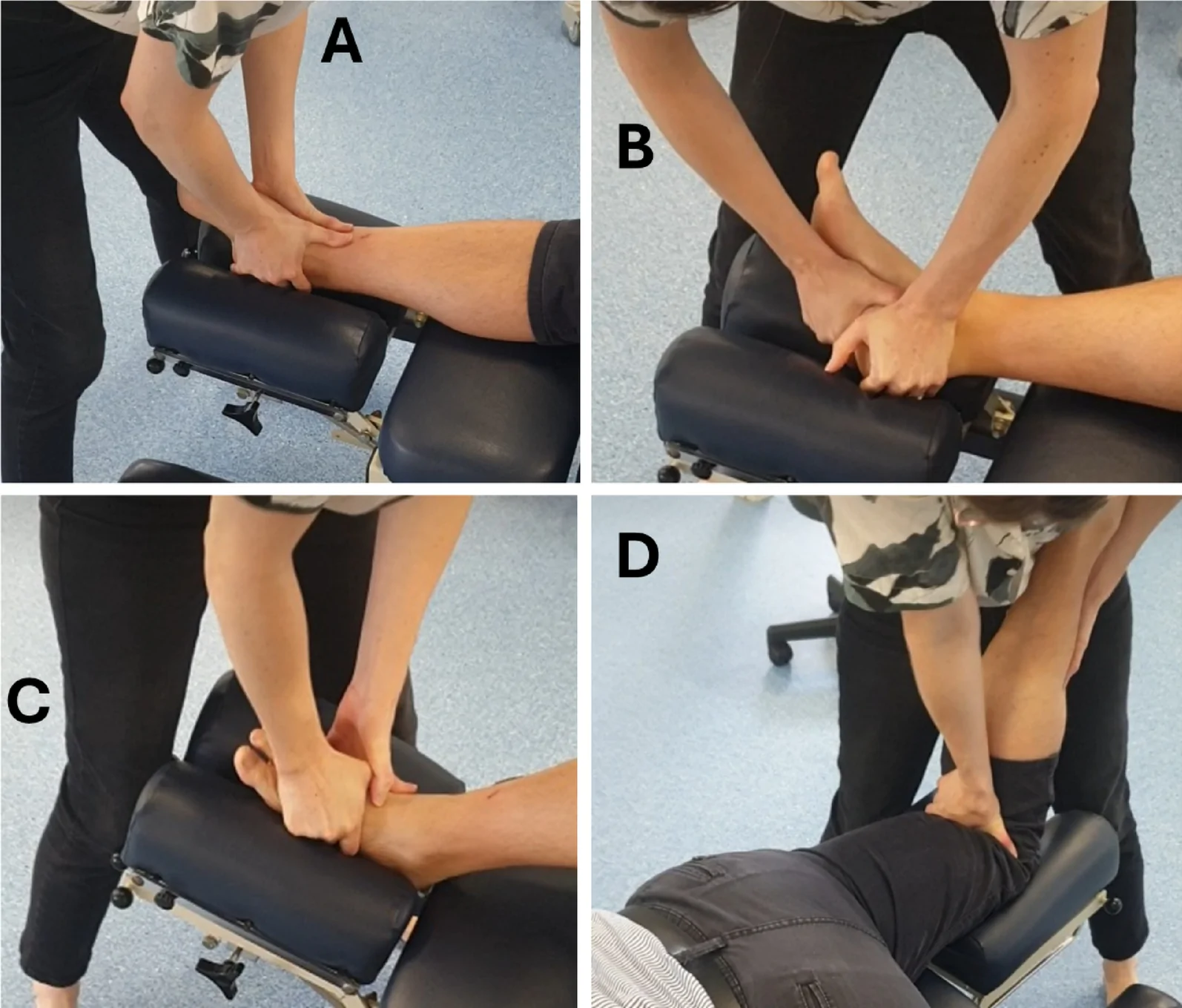
Sham manipulations. **a** Talocrural joint anterior to posterior, **b** Subtalar joint medial to lateral, **c** Tarsometatarsal joint anterior to posterior, **d** Proximal Tibiofibular joint posterior to anterior

The sham manipulations consisted of mechanically assisted mock thrusts using the drop mechanism of a treatment table to multiple joints bilaterally, as seen in Fig. 4. Each manoeuvre was intended to mimic a real manipulation but involved broad contact and for most the mock thrust was delivered into the table to trigger the drop mechanism, rather than into a joint. Each was repeated 2–3 times to enhance the perception of treatment being delivered, and as is typical when drop-piece manipulations are used in clinical practice. The drop mechanism was chosen as the sound of the dropping mechanism and sense of movement would help mask the lack of audible cavitations or joint thrust.

### Outcome measures

**Fig. 5:**
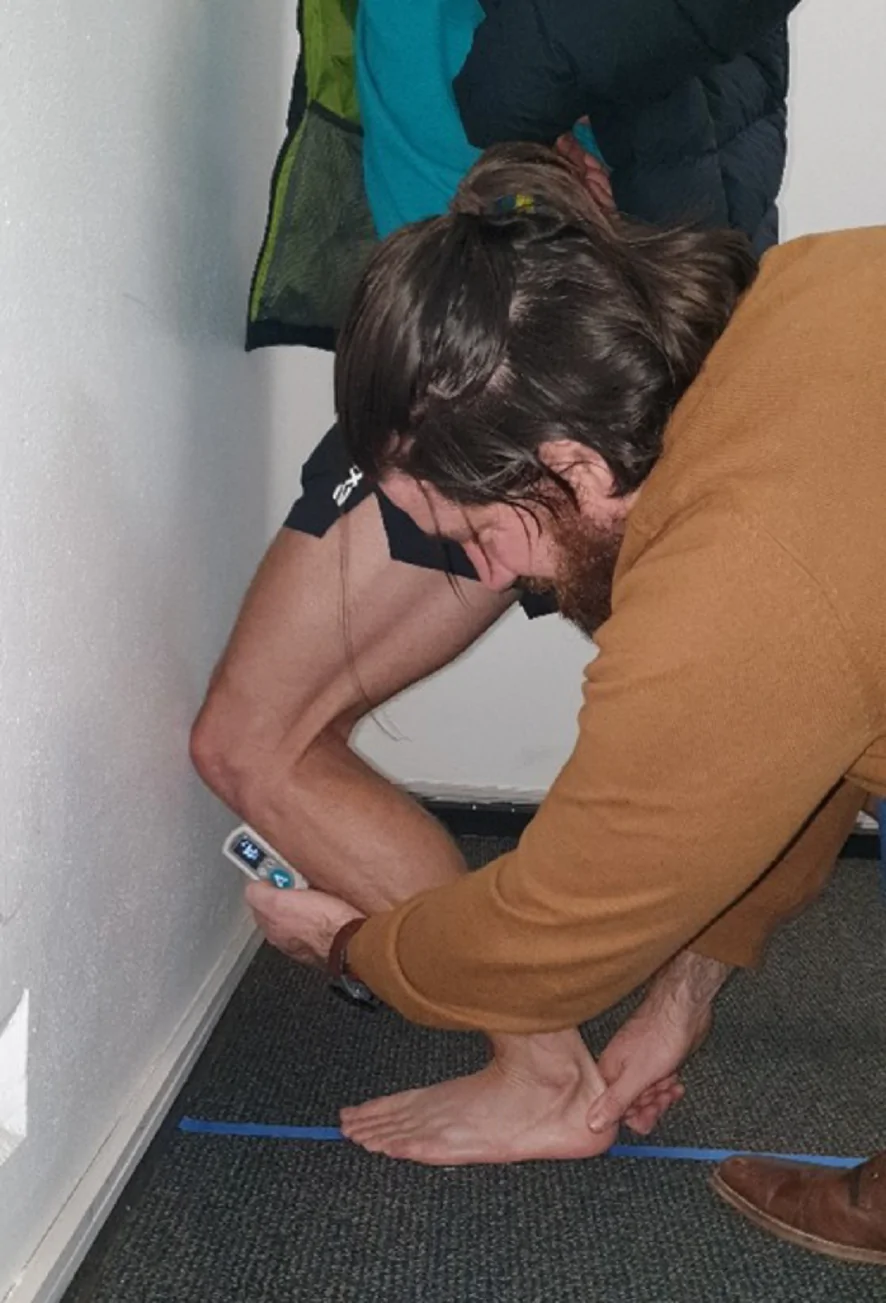
Weightbearing lunge test with digital goniometer at end range of motion

The primary outcome measure was the 1RM back squat weight (kg). The secondary outcome measures were maximal DF-ROM during the 1RM squat (degrees), measured using the 3D motion capture software, and active DF-ROM (degrees), measured using the WBLT. The WBLT was measured according to the method outlined by Dill et al. [18] using a digital goniometer (EasyAngle® Meloq AB, Sweden). See Fig. 5. This was repeated three times for each ankle and the average used. This method has good intra-rater (ICC = 0.98, 95% CI = 0.93–0.99) and inter-rater reliability (ICC = 0.97, 95% CI = 0.90–0.99) [43].

### Data analysis

Kinematic data was processed using Visual 3D software (Version 5, C-Motion, Germantown, USA). Raw trajectories were filtered using a 6 Hz low pass Butterworth filter. Functional hip joint centres and knee flexion extension axes were identified [52]. A standard joint coordinate system was used to calculate joint angles at the ankle with plantar/dorsi flexion calculated as the rotation of the foot s around the medial lateral axis of the tibial coordinate system [53].

Statistical analyses were performed using SPSS version 25.0 (IBM Corporation, Armonk, NY, USA). Means and standard deviations, or frequencies, are presented for baseline variables. For the primary outcome of 1RM weight, a one-way repeated measures ANCOVA was performed with the within-subject factor of time (baseline, post-manipulation, and post-sham). For DF-ROM during the squat, a two-way repeated measures ANCOVA was performed using within-subjects factors of time (post-manipulation and post-sham) and ankle (less restricted ankle and more restricted ankle). For active DF-ROM, a two-way repeated measures ANCOVA was performed using within-subjects factors of time (baseline, post-manipulation, and post-sham) and ankle (less restricted ankle and more restricted ankle). For this ANCOVA, change in ankle DF-ROM from pre to post warm-up was used as the outcome measure to account for the effect of warm-up on ROM and more accurately assess the impact of treatment. All ANCOVAs included intervention sequence as a covariate. This deviates from the a-priori statistical plan, which was to use independent T-tests to compare interventions, as an ANCOVA allowed the inclusion of intervention sequence as a covariate as recommended by the CONSORT guidelines [54].

Assumptions were checked prior to running ANCOVAs. Using the Shapiro–Wilk test, a small number of outcomes at specific time points were not normally distributed, however, ANOVAs are known to be robust to deviations from normality [55], so we chose to proceed. Mauchly’s test of sphericity indicated that the assumption of sphericity was not violated for each ANCOVA. Statistical significance was set at *p* ≤ 0.05. Estimated marginal means, standard deviations, and 95% confidence intervals are presented for the results of the ANCOVAs. Post-hoc tests were performed with Sidak adjustment where indicated [56].

Effect sizes for within-group changes in 1RM back squat were calculated using Hedges’ g, which adjusts for small bias and is appropriate for evaluating the magnitude of within-condition changes. Effect sizes were interpreted using conventional thresholds, where values of 0.2, 0.5, and 0.8 are considered small, medium and large, respectively [57].

Blinding was assessed using the James’ and Bang’s Blinding Index (BI) in R studio v. 2021.09.0 [58] using the BI package. The BI’s were calculated according to Bang et al. [59]. For James’ BI (overall blinding success), a value of 0.5 to 1 indicates successful blinding. For Bang’s BI (per treatment arm blinding success), we considered a scenario of “random guess” (BI = − 0.2 to + 0.2 for each arm) or “wishful thinking” (roughly equal but opposite BI i.e., + x for the real intervention and -x for the sham) as indicating successful blinding [60].

## Results

**Fig. 6:**
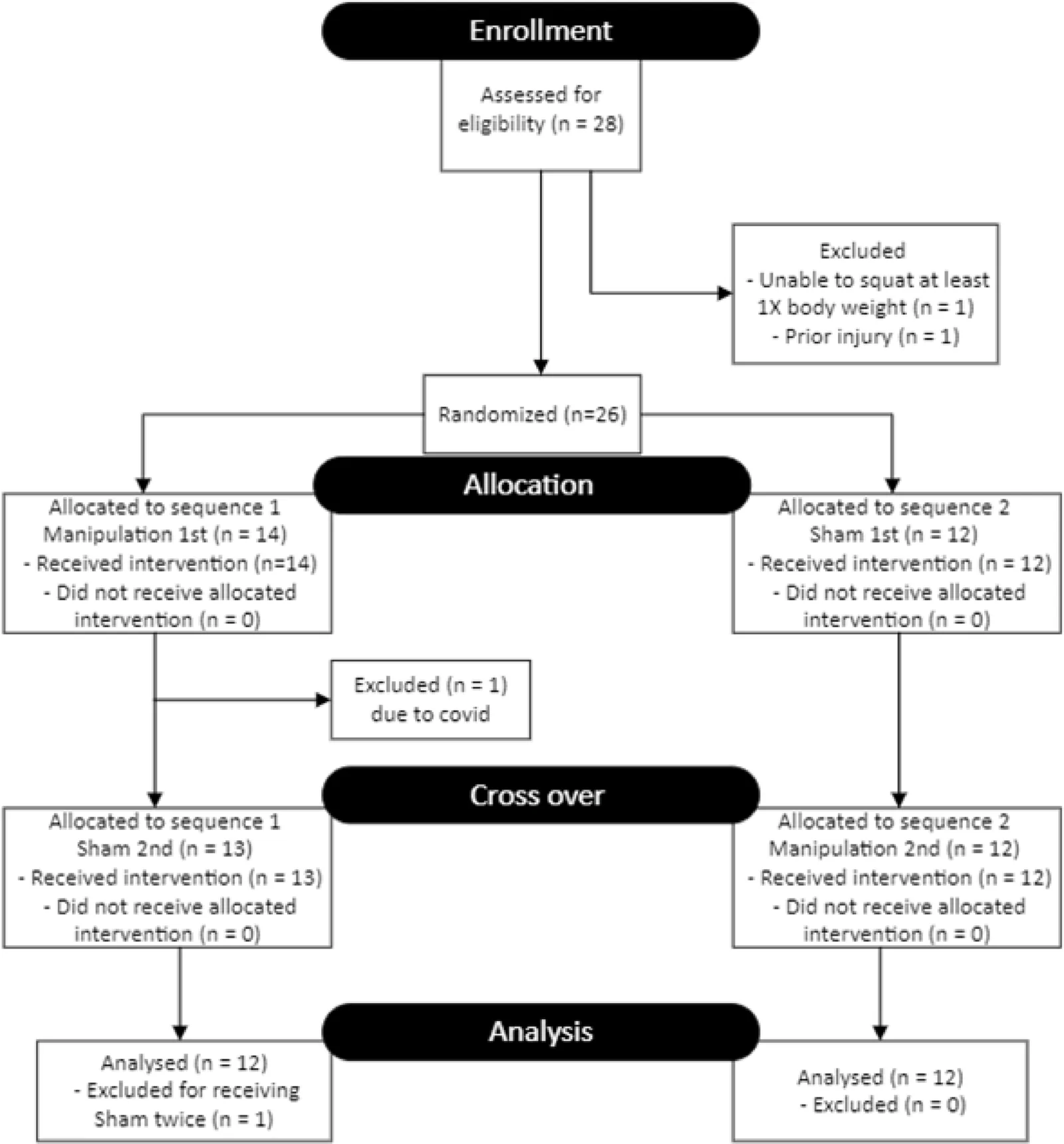
Participant flow chart

Twenty-six people participated in the study between July 2022 and March 2023. Two participants were excluded from analyses after randomisation; one was unable to complete the study due to illness and one was excluded due to an administrative error resulting in them receiving the sham intervention twice. See Fig. 6 for the participant flow chart. The participants had a mean age of 29.2 years (± 5.7) with 17 males and 7 females. There were no major differences in baseline characteristics between sequences, detailed in Table 1. There were no reported adverse events during or after the trial. An average of 1.8 thrust attempts per joint was required to achieve perceived success for the HVLA manipulations. Furthermore, there was no significant difference in the average number of attempts required between the two therapists (therapist 1: 2.2 attempts; therapist 2: 1.6 attempts; *p* = 0.075).

**Table 1:** Baseline participant characteristics (mean ± SD)

Variables	Sequence 1	Sequence 2
Participants (n)	12	12
Male (n, %)	8, 66	9, 75
Female (n, %)	4, 33	3, 25
Age (y)	30 ± 6.8	28.5 ± 4.5
Height (m)	1.74 ± 0.07	1.78 ± 0.01
Weight (kg)	83.7 ± 21.0	88.7 ± 20.3
1RM back squat (kg)	132.4 ± 44.7	128.1 ± 48.1
Relative 1RM Back Squat (kg^.^kg^−1^)	1.6 ± 0.3	1.4 ± 0.4
*Ankle dorsiflexion range of motion (°)*		
More Restricted Ankle (°)	30.6 ± 4.9	32.4 ± 7.8
Less Restricted Ankle (°)	35.0 ± 5.0	36.5 ± 9.0
FADI (%)	99.0 ± 2.8	96.6 ± 6.1

SD = standard deviation, n = number, Less Restricted Ankle = the ankle having the greatest dorsiflexion range of motion at baseline, More Restricted Ankle = the ankle having the lowest dorsiflexion range of motion at baseline; FADI = Foot and Ankle Disability Index

### 1RM back squat

The 1RM data was normally distributed at each time point as assessed by the Shapiro–Wilk test (all *p* ≥ 0.33), and all other assumptions were met. The one-way repeated measures ANCOVA on back squat 1RM was statistically significant (*p* = 0.02, partial η^2^ = 0.16), but subsequent Pairwise comparisons indicated there were no statistically significant between-group differences (mean difference = 2.00 kg, 95% CI = − 0.93 to 4.99,* p* = 0.27). This indicates that while there was an overall improvement in 1RM strength from baseline across the sessions, this was not a treatment-specific effect. The estimated marginal mean 1RM back squat at baseline was 130.2 kg (95% CI: 110.6–149.9, SD = 45.5). Following the sham, the mean 1RM increased by 2.74 kg (95% CI: 0.19–5.31, *p* = 0.032), with a negligible effect size (Hedges’ g = 0.087). After the manipulation, the mean 1RM increased by 4.75 kg (95% CI: 2.32–7.18, *p* =  < 0.001), with a small effect size (Hedges’ g = 0.143).

**Fig. 7:**
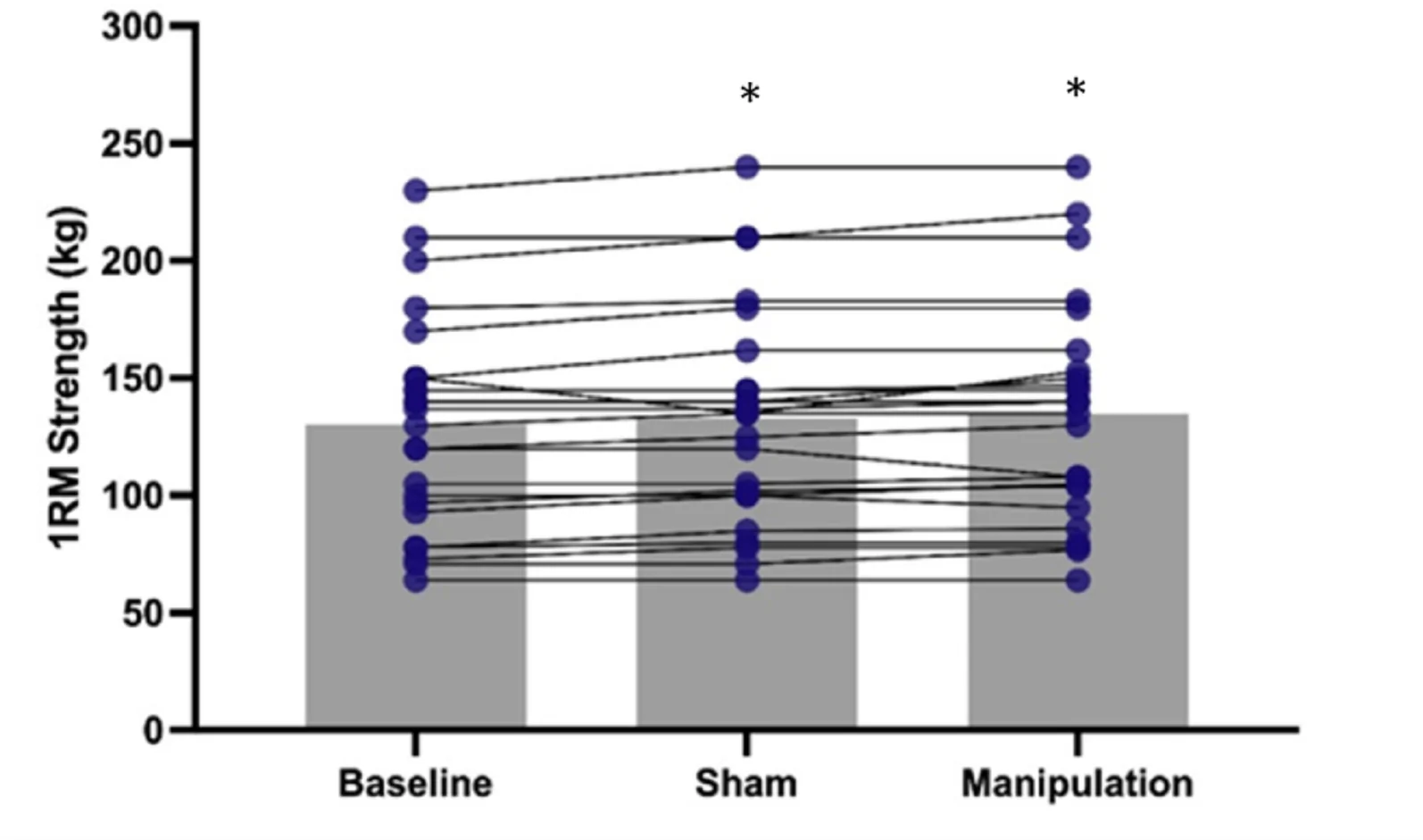
1RM barbell back squat means at each session and individual participant change

See Table 2 for estimated marginal means and Fig. 7 for group means bars and individual participant changes.

**Table 2:** Estimated marginal means per time point

Time point	1RM (kg)	95% CI of 1RM	SD	Change from baseline (Kg)	95% CI of change from baseline	Change from baseline *p* value	Effect size
Baseline	130.2	110.6–149.9	45.5	–	–	–	
Post-sham	133.0	112.8–153.2	46.7	2.74	0.19–5.31	0.032	0.087
Post-manipulation	135.0	114.5–155.5	47.5	4.75	2.32–7.18	< 0.001	0.143

Effect sizes are reported as Hedges g

### Dorsiflexion range of motion during squat

Five participants were excluded from kinematic analyses due to missing markers during motion capture. The data was normally distributed (all *p* > 0.05), and all other assumptions were met. The two-way repeated measures ANCOVA on maximal ankle dorsiflexion during a 1RM back squat was not statistically significant (*p* = 0.239, partial η^2^ = 0.21).

### Dorsiflexion range of motion

The ankle DF-ROM during the WBLT was normally distributed (all *p* ≥ *0.104)* except for at the sham session on the more restricted ankle in sequence 1 (*p* = 0.046), and all other assumptions were met. The two-way repeated measures ANCOVA examining ankle DF-ROM was not statistically significant (*p* = 0.39, partial η^2^ = 0.037). See Table 3 for estimated marginal means.

**Table 3:** Estimated marginal means for ankle DF-ROM during WBLT per intervention

	Maximal DF-ROM, mean	SD	95% CI
*More restricted ankle (º)*			
Baseline	31.8	1.3	29.1–34.5
Manipulation	34.7	1.4	31.8–37.6
Sham	35.6	1.3	33.0–38.2
*Less restricted ankle (º)*			
Baseline	35.3	1.4	32.3–38.2
Manipulation	36.6	1.3	33.8–39.3
Sham	37.6	1.5	34.3–40.8

### Blinding

The James’ BI indicated that overall blinding was adequate, being close to a random guess scenario (0.53, 95% CI = 0.43–0.63). Using the Bang’s BI to assess each intervention arm indicated blinding was verging on the wishful thinking scenario [60] but there were differences in blinding for each intervention. The participants mostly guessed they received a real intervention correctly, indicated by the manipulation BI approaching one (Bang’s BI = 0.96, 95% CI 0.88–1.00). For the sham intervention, participants tended to guess they received a real intervention (Bang’s BI = − 0.50, 95% CI − 0.76 to − 0.24), indicated by the negative BI, but the magnitude was not equivalent between the intervention arms. Therefore, the study had overall effective blinding but with some imbalance between interventions.

## Discussion

The findings of this study do not support our hypothesis that a single session of ankle and knee manipulations would increase immediate 1RM back squat strength and improve ankle DF-ROM.

### Back squat 1RM

There were no significant differences between interventions for change in back squat 1RM. There was a statistically significant increase in back squat 1RM of 4.75 kg (3.6%) after manipulation and 2.75 kg (2.1%) after sham treatment, however, these changes are likely not practically relevant given there was no significant difference between interventions. In trained cohorts, a practically relevant improvement is defined as a change that systematically exceeds both random measurement error and normal test-test variation. The estimated normal between session 1RM variance for the 1RM back squat can reach 4.8% in trained men and between 2 and 4% in trained women [49, 61]. Furthermore, an estimated absolute minimal detectable change at a 95% confidence level for the 1RM back squat in well-trained athletes has been estimated at approximately 4.8 kg [62]. Because our observed acute increase after the manipulation falls below both the expected relative variance threshold (2–4.8%) and the absolute minimal detectable change threshold (4.8 kg), it does not meet the criteria for practical relevance.

This interpretation is supported by the small effect sizes observed (Hedges’ g = 0.14 for manipulations and 0.09 for sham). [49] Hence, the changes in squat strength observed in this study may be a result of normal day to day variation, a learning effect from repeated exposures, a placebo effect [63], or a combination. In addition, resistance-trained individuals, who already possess high levels of strength and fitness, may experience a ceiling effect, limiting the potential for improvements over short time periods [40].

While there does not appear to be any similar studies investigating the effect of lower limb manipulations on back squat performance, our findings are similar to a pilot study in handball players with talocrural joint dysfunction [37]. Hedlund et al. [37] reported no significant difference in vertical jump height after talocrural manipulation once a week for three weeks compared to sham, though there was an increase from baseline in the manipulation group [37]. Additionally, in a single-arm study on participants with chronic ankle instability, no significant changes in maximal isometric contraction force of the tensor fascia latae, gluteus medius, and rectus femoris were observed immediately following manipulations of the talocrural, subtalar, proximal, and distal tibiofibular joints [34]. However, there was a significant increase 48 h post manipulation in gluteus medius activity [34], suggesting that there may be delayed treatment effects. In the realm of sports and clinical outcomes, the presence of pain can impede an individual’s ability to engage in movement-based strategies. Pain can result in motor inhibition, altered movement patterns, and impairments in sport-related activities like running and jumping [64]; therefore, it is possible that the effect of lower limb manipulations on squat performance may be different in a symptomatic population. Furthermore, it may take multiple intervention sessions to elicit change in an athletic population e.g., over at least three weeks [27, 29, 30, 32].

### Dorsiflexion during back squat

The observed non-significant difference in maximal ankle DF-ROM during a 1RM back squat following the real or sham intervention may be because squat depth height was limited to parallel and participants were allowed to wear shoes with a heel lift, which allows for increased anterior tibial translation without changes to the required ankle DF-ROM [38, 39]. The effect of manual therapy interventions such as manipulation or mobilisation on ankle DF-ROM during a maximal back squat had not been tested in any prior studies.

### Dorsiflexion during weightbearing lunge test

The observation of no significant changes to ankle DF-ROM during the WBLT in this study are consistent with several studies on the effect of lower limb manipulations on DF-ROM in participants who have lateral ankle ligament strains or chronic ankle instability [26, 28, 31]. However, these studies all used a non-weightbearing ROM measurement method, compared to the WBLT used in this study.

The studies by Ritter [24] and Marrón-Gómez et al. [25] used the WBLT, however, both reported significant increases in ankle DF-ROM following a single intervention including lower limb manipulations. The conflicting results between this study and the previous studies using the WBLT may be explained by differences in populations. This study recruited resistance-trained participants with asymptomatic loss of ankle DF-ROM, while Ritter [24] recruited asymptomatic participants with a motion restriction and/or asymmetry in motion between the same joints of opposite legs and unknown training history, and Marrón-Gómez et al. [25] recruited participants with chronic ankle instability from university soccer and basketball teams.

The most notable methodological difference in this study compared to previous studies with lower limb manipulation is the use of a sham with a mock thrust. Few similar studies compare lower limb manipulation to sham, and none use a sham that attempts to replicate the active intervention. Pellow and Brantingham [29] compared multiple sessions of talocrural manipulation to a detuned ultrasound placebo and found that DF-ROM increased significantly after manipulations one month following the interventions compared to the placebo. This suggests that manipulations may not affect ankle DF-ROM immediately following interventions in a resistance-trained population, but changes may occur after multiple interventions.

### Blinding

The majority of participants believed both interventions were real based on the Bang’s BI, however this belief was somewhat less likely after the sham compared to the HVLA manipulation. The small imbalance in blinding may indicate modestly differing expectations after each intervention, which may have impacted participant’s effort during outcome testing. This may have been associated with the forces applied to the joint, which were intentionally less during the sham and this may have unblinded participants [65]. This is indicative of the difficulty of providing an adequate sham in manual therapies [65]. Another contributor may have been that participants were not required to be naïve to joint manipulation, and joint manipulation may be highly recognisable by participants who have prior experience with it [66]. Additionally, these participants may associate an audible cavitation with success and a lack of cavitation as unsuccessful [66]. While the sham intervention attempted to mask the lack of cavitations with the use of the mechanical drop-piece, the expectation of a cavitation may still have influenced their perceptions.

## Limitations

The current study had several limitations. Firstly, the sham has not been validated. While the therapists applying the sham were careful to apply minimal pressure directly to joints during the sham and the thrust was largely delivered into the table to trigger the mechanical drop mechanism, it is possible the sham elicited a therapeutic effect through movement of the joint, stretching of surrounding tissues, and stimulation of mechanoreceptors [67, 68]. Any element within the placebo that enhances the therapeutic impact of the control procedure can decrease the effect size of the trial, leading to a type-II error. Therefore, it is possible that the use of the sham with a mock thrust could have influenced the study’s outcome [66].

Secondly, the inclusion criteria of a minimum squat strength of one-times body weight may have attracted a population with less learned movement and muscle recruitment patterns compared to a population that can squat higher weights. Additionally, although this study was designed to be similar to the participant’s normal training practices, participants may have changed their lifting behaviours to accommodate squatting inside of a squat cage and using spotting rails set just below parallel. The observed improvements in 1RM squat strength may have been due to a learning effect, or due to the participants feeling eager to perform.

Finally, allowing the use of ≤ 1inch heel lifts in this study may have changed squat movement biomechanics, potentially influencing distribution of forces and the activation of different muscle groups [69, 70]. Using a heel lift may allow individuals with limited ankle mobility to squat deeper than they would be able to without the lifts [39, 69, 71, 72]. Consequently, this could have influenced the observed effects of the ankle and knee manipulations on ankle DF-ROM during the back squat strength.

### Future research

In future studies examining the effect of lower limb manipulation, researchers should consider using a validated sham technique. Overall, it appears that studies investigating only immediate effects of a single session of lower limb manipulation on squat strength and kinematics may not provide additional insight in asymptomatic, well-trained cohorts. However, delivering multiple intervention sessions may reveal cumulative differences in arthrokinematics, tissue compliance, or neural drive that might occur over longer time periods. The addition of surface electromyography to determine if HVLA manipulations influence motor unit recruitment patterns, a neurophysiological pathway that may be altered even in the absence of overt mechanical changes of the primary muscle groups, may also be valuable [23]. To further understand the clinical relevance of lower limb manipulation in individuals with ankle DF-ROM deficits, it would be valuable to investigate a population with chronic ankle instability in future studies. This population presents with arthogenic muscle inhibition and impaired sensorimotor control [35]. In such populations, HVLA manipulation is theorised to act via a ‘disinhibition’ mechanism, restoring sensory afferent input to facilitate motor neuron pool excitability [33], which may translate more directly to improved maximal strength outcomes than in asymptomatic participants.

### Practical applications

For sports chiropractors, physical therapists, and strength and conditioning professionals, these findings provide important clinical context regarding the efficiency of manual therapy in athletic settings. Specifically, clinicians should use lower limb manipulative therapy with discretion when preparing asymptomatic, well-trained athletes for maximal strength testing (1RM) in the back squat to a parallel depth. While a small acute effect was observed following the intervention, resolving a localised mechanical restriction (DF-ROM deficit) did not translate to practically relevant improvements that exceeded normal test-to-test variance. Therefore, passive joint manipulation should not be expected to acutely increase maximal neural drive or serve as a primary driver of maximal force output during competition or testing. However, manipulative therapy may still be applied with discretion during general maximal strength training cycles as a preparatory adjunct, provided it is integrated with active motor control and heavy resistance training.

## Conclusions

High-velocity low-amplitude ankle and knee manipulations did not increase back squat 1 repetition maximum strength short-term compared to a sham treatment in a resistance-trained population with asymptomatic ankle dorsiflexion range of motion restrictions. The manipulations also had no effect on maximal ankle dorsiflexion during the squat, or dorsiflexion range of motion.

## Data Availability

Anonymised data summarising 1RM and ankle DF-ROM available on request from the corresponding author.

## Abbreviations

DF-ROM: Dorsiflexion range of motion
HVLA: High velocity low amplitude
1RM: 1 Repetition maximum
ROM: Range of motion
WBLT: Weightbearing lunge test
